# The Cds.71 on *TMS5* May Act as a Mutation Hotspot to Originate a TGMS Trait in Indica Rice Cultivars

**DOI:** 10.3389/fpls.2020.01189

**Published:** 2020-08-07

**Authors:** Yanning Tan, Xuewu Sun, Baohua Fang, Xiabing Sheng, Zheli Li, Zhizhong Sun, Dong Yu, Hai Liu, Ling Liu, Meijuan Duan, Dingyang Yuan

**Affiliations:** ^1^State Key Laboratory of Hybrid Rice, Hunan Hybrid Rice Research Center, Changsha, China; ^2^State Key Laboratory of Hybrid Rice, Hunan Academy of Agricultural Sciences, Changsha, China; ^3^Hunan Rice Research Institute, Hunan Academy of Agricultural Sciences, Changsha, China; ^4^College of Bioscience and Biotechnology, Hunan Agricultural University, Changsha, China; ^5^College of Agronomy, Hunan Agricultural University, Changsha, China; ^6^Long Ping Branch, Graduate School of Hunan University, Changsha, China

**Keywords:** rice (*Oryza sativa* L.), two-line hybrid rice, thermo-sensitive genic male sterility gene 5 (*tms5*), thermo-sensitive genic male sterility (TGMS), mutation hotspot, T98S, unbalanced allele frequency, indica

## Abstract

The gene *tms5*, which controls thermo-sensitive genic male sterility (TGMS), has been widely used in two-line hybrid rice breeding in China. The *tms5* lines have two sources, namely, AnnongS-1 (AnS) and Zhu1S (ZhS) and, interestingly, are commonly subject to an alteration at cds.71. However, whether cds.71 acts as a mutation hotspot is unknown. Herein, another *tms5* mutant named T98S (induced from T98B by irradiation) was used to explore this. First, the gene of *tms(t)* responsible for T98S was fine-mapped on chromosome 2 based on an F_2_ group of T98S/R893. In T98S, the candidate gene *TMS5* (LOC_Os02g12290.1) mutated at cds.71 with a transversion from cytosine (C) to adenine (A), as also observed in AnS and ZhS. Moreover, the entire coding sequence of *TMS5* from T98B converted T98S from sterile to fertile by *Agrobacterium tumefaciens*-mediated transformation, confirming that T98S is controlled by *tms5*. Next, detection on nearly 40,000 single nucleotide polymorphisms (SNPs) on Rice 56K SNP Array revealed T98S was 99.99% similar to T98B but only 72.84% and 77.47% similar to AnS and ZhS, respectively, demonstrating that T98S originated from T98B rather than from existing *tms5* lines. Furthermore, the cds.70 was found to exist as a T/G haplotype, and it was T rather than G that helped to induce a TGMS trait. The T frequency was 67.52% in indica rice but decreased to 1.75% in japonica rice in 2,644 cultivars tested, which partly explains why *tms5* mutants were mostly found in indica lines. Our findings provide evidence that cds.71 may act as a mutation hotspot and clues for breeding TGMS lines in a more efficient way.

## Introduction

Improving rice yields has become increasingly urgent due to human population growth, particularly in Asia and Africa. Rice is a crop with strong heterosis of ~ 10% in yield(Li et al., 2013). The heritable trait of male sterility is favored in breeding hybrid rice with strong heterosis and in developing hybrid seeds for commercial use (Yuan, 1966). Hybrid rice bred *via* a three-line system is based on cytoplasmic-nuclear male sterile lines (CMS lines) in China before the 1990s (Yuan, 1997). The male sterility of a CMS line is retained by a maintainer line and recovered by a restorer line (Yuan, 1997), thereby increasing the labor input in the production of hybrid rice seeds.

To simplify the hybridization procedure, a two-line system was successfully established in the late 1980s that utilizes either photo- or temperature-sensitive genic male sterile lines (P/TGMS lines) (Shi, 1985; Yang et al., 1993; Deng et al., 1999). The P/TGMS lines, because of their control *via* recessive nuclear gene(s), can be recovered by almost any rice cultivar. This greatly improves the hybrid rice breeding efficiency (Huang et al., 2014). In particular, the pollen fertility in P/TGMS lines can be shifted with environmental conditions. This allows them to be pollinated by restoring lines to produce hybrid seeds when sterile and to be self-pollinated for reproduction when fertile. The P/TGMS lines are typically divided into two basic ecotypes, PGMS and TGMS (Yuan, 1990). The PGMS lines in application are derived from a japonica rice cultivar Nongken58S and convert sterile to fertile under a short day-length and low temperature (Shi, 1985; Yuan, 1997). It was found that Nongken58S is determined by several genes, and the dominant one is *pms3* (also named as *p/tms12-1* in indica rice cultivar Peiai 64S*)* on chromosome 12. The *pms3* gene encodes a long noncoding RNA that may be involved in epigenetic modifications to influence pollen development (Ding J. et al., 2012; Zhou et al., 2012). Such a complicated manipulation on pollen fertility has been greatly improved in TGMS lines. The TGMS lines are restricted solely by temperature and fail to produce pollen grains under high temperature (Chen et al., 1994; Yang et al., 2000; Lee et al., 2005), thus rendering them useful in hybrid production from a safety perspective. Since 2012, the cultivation areas and numbers of TGMS line-based hybrid combinations have exceeded 95% in two-line hybrids, contributing greatly towards food security in China (Zhang et al., 2015).

A practical TGMS line is derived either from Annong S-1 (AnS) or Zhu-1S (ZhS), which are well-known early-season indica rice cultivars in south China. As reported, AnS and ZhS originated independently; AnS was a spontaneous mutant discovered in Annong, and Annong was obtained from a population of a triple cross of Chao40B/H285//6209-3 (Deng et al., 1999). The line ZhS emerged from a F_2_ male sterile plant of Ke-fu-2/Xiang-zao-xian 3//02428 (Yang et al., 2000). Interestingly, both AnS and ZhS were found to be controlled by *tms5* (*thermo-sensitive genic male sterile 5*, *tms5*) on chromosome 2 (chr.2) and are commonly subjected to a point altered at cds.71 (Wang et al., 2003; Yang et al., 2007; Sheng et al., 2013; Zhou et al., 2014). In normal fertile rice cultivars, *TMS5* encodes a functional nuclear ribonuclease Z (RNase Z) to process *Ub_L40_* mRNA into pieces, which is presumed to be a prerequisite for pollen development (Zhou et al., 2014). In contrast, in AnS and ZhS, the change from C to A at cds.71 terminates transcription in advance and accordingly produces a loss-of-function RNase Z to create a TGMS trait (Zhou et al., 2014).

Gene mutation is a common phenomenon in organisms, and the sites on a gene are not equally mutable. The points that mutate at a higher frequency are often called “mutation hotspots” (Coulondre et al., 1978) and are valuable resources to explore the mechanisms underlying gene mutation. Based on the same mechanism underlying both AnS and ZhS, we hypothesize that the cds.71 on *TMS5* act as a mutation hotspot to induce a TGMS trait. To support this, additional *tms5* mutants from diverse genetic backgrounds were screened. We identified a TGMS mutant named T98S that is a ^60^C_O_-γ irradiation-induced mutant at M_2_ from T98B (an elite indica maintainer for three-line hybrid rice in China) (Tan et al., 2018). Observations on morphological specificity revealed that T98S was very similar to T98B in plant shape, yield factors, and flowering habits. Furthermore, T98S was found to be controlled by a recessive nuclear gene, and it presented sterile at 28.0°C and fertile at 22.0°C regardless of the day-length being adjusted to 12.0 h or 13.5 h in the chamber (Tan et al., 2018). Although T98S is quite similar to the TGMS lines AnS and ZhuS in fertility, whether it is controlled by *tms5* remains unknown. Here, using a series of genetic analyses, we confirm that T98S is another *tms5* line with a possible hotspot at cds.71 and explore further why the *tms5* lines were mostly found in indica rice cultivars.

## Materials and Methods

### Materials

The line T98B is an indica maintainer of wild-type CMS line T98A (Deng et al., 2001). The line T98S is a TGMS mutant identified from T98B through ^60^Co-γ irradiation (Tan et al., 2018). The two indica TGMS lines, AnnongS-1 (ANS) and Zhu1S (ZhS), were reportedly bred from different sources but are commonly controlled by *tms5* (Zhou et al., 2014). These materials were all provided by the Hunan Hybrid Rice Research Center, China.

### Mapping the Target Gene for T98S

The bulked-segregant analysis (BSA) method was used to rough map the target gene *tms(t)* responsible for T98S (Michelmore et al., 1991). An F_2_ population from T98S crossed with R893 (an indica rice with normal pollen fertility) was established. Twenty-five F_2_ sterile/fertile individuals were separately collected to extract DNA using CTAB buffer (McCouch et al., 1988) and establish two bulked DNA samples. Then, 125 polymorphic simple sequence repeat (SSR) markers covering 12 rice chromosomes (http://www.Gramene.org) were used to test the genotype difference between TGMS-plants DNA pool and T98S by polyacrylamide gel eletrophoresis. Finding associated markers which showed similar genotype between the two samples and determining chromosomal location of *tms(t)*. Later, *tms(t)* was fine mapped following the method of linkage analysis (Qi et al., 2014). In the rough mapping region, developing plenty of polymorphic insert-deletion (InDel) markers and sequence-tagged site markers provided by the Rice SNP-Seek Database (https://snp-seek.irri.org/). Subsequently, 1334 F_2_ TGMS individuals were used to identify more closely linked markers and fine map *tms(t)* based on the number of the recombinants. Finally, the candidate of *tms(t)* in the mapping region was deduced based on the database of the Rice Genome Annotation Project (RGAP, http://rice.plantbiology.msu.edu/index.shtml). Primer information is listed in **Supplemental Table S1**.

### Validation of Whether *tms5* Is Responsible for T98S

The full coding sequence (CDS) of *TMS5* (909 bp) cloned from T98B was inserted in the vector pTMS5, which was driven by the maize *ubiquitin* promoter and terminated at the terminator of nopaline synthase gene (*NOS*) from *Agrobacterium tumefaciens*. This construct was then introduced into the callus of T98S induced from mature seeds by *Agrobacterium tumefaciens*-mediated transformation (Holsters et al., 1978). The transgenic plants were detected *via* PCR with specific primers ubi-F/tms-R (listed in **Supplemental Table S1**). The tested materials including homozygous T_1_ lines, T98S (negative control) and T98B (positive control), were transplanted (with 30 individuals for each line) at one batch in the paddy field at Changsha, China, on May 25^th^, 2018. The young seedlings undergoing the fertility sensitive phases from IV to VI of young panicle differentiation (July 5^th^–12^th^) were treated to heat stress under a daily mean temperature of 29°C. Five panicles of different individuals from each line from July 11^th^ –19^th^ were measured for the ratio of stained ring pollen grains (fertile pollen grains) treated with 3.0% I_2_-KI under an optical microscope stain. The values (mean ± sd) were analyzed using one-way analysis of variance (ANOVA) at 0.05 significance level.

### Detection of the Genetic Background for T98S

DNA was extracted from the leaves of T98B and T98S following to the CTAB method. The DNA quality was assessed for quantity (≥1.5 µg), concentration (≥30 ng/µl) and purity (ratio of 260 nm/280 nm = 1.8–2.0) with NanoDrop. And then the resultant DNA was visualized on agarose gel for integrity control.

DNA genotyping was performed on Rice 56K SNP Array (Huazhi Biotech Com. LTD). Initially, the DNA was amplified and fragmented. Prior to DNA hybridization, the fragments were purified followed by chip hybridization, scanning and subsequently data quality control. The clean genotyping data (call rate≥97%) with high resolution and polymorphism was further used to calculate the homozygosity and genetic difference between samples. The loci with identical genotypes for three replicates between two samples are assigned to “identical loci” (IL), while the loci with different genotypes between them are assigned to “polymorphic and homozygous loci” (PHL-1) or “polymorphic and heterozygous loci” (PHL-2). The genetic similarity and genetic difference rate between two samples are calculated with the formulas: genetic similarity = (the number of IL)/(total number of loci) *100%; genetic difference rate = (the number of PHL-1 and PHL-2)/(total number of loci) *100%. Accordingly, the variation between two pairs of samples (“T98S” and “AnS”; “T98S” and “ZhS”) were respectively evaluated.

In addition, the genetic background of T98S was compared with AnS and ZhS by the same above procedure. The background difference rate equals the ratio of polymorphous SNP numbers to total SNPs. In particular, a region of about 1.0 Mb around *TMS5* (5.39–7.39 M) was studied to identify specific SNPs for T98S.

### Allele Frequency Analysis of Cds.70 Among Rice Cultivars

To explain why the original *tms5* mutants tends to be discovered in indica rice rather than other groups, the genotype of cds.70–72 (from 6,397,411 to 6397413 bp on chr.2) was analyzed among rice cultivars obtained from the Rice SNP-Seek Database (https://snp-seek.irri.org/) (Wang et al., 2018). The allele frequencies of cds.70 (T) and cds.70 (G) were investigated for indica cultivars (including subpopulations of indx, ind1A, ind1B, ind2, and ind3) and japonica cultivars (including subpopulations of jap, temp, subtrop, and trop). Furthermore, the changes in the TMS5 domains were assessed on whether the genotype of cds.70–71 was altered from GC to GA, or from TC to TA by CD-Search on the NCBI platform (https://www.ncbi.nlm.nih.gov/Structure/cdd/wrpsb.cgi).

## Results

### *TMS5* Mutated at Cds.71 in T98S

**Figure 1:**
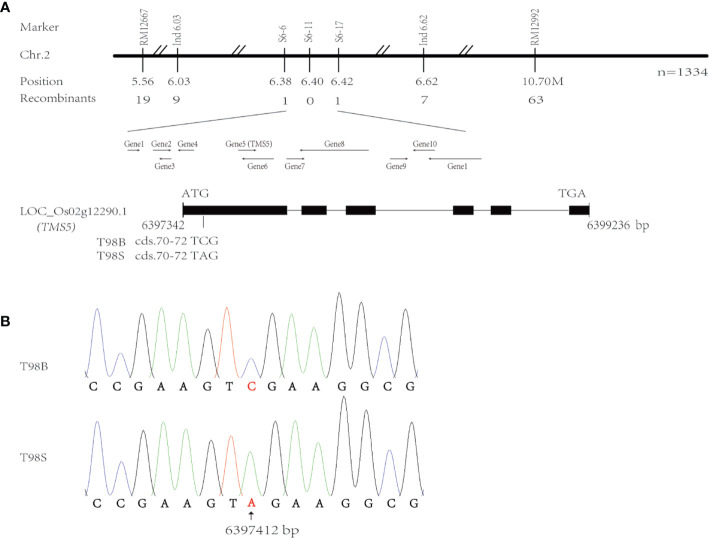
Analysis of candidate genes for T98S in fertility. **(A)** Fine-mapping the *tms(t)* responsible for T98S on chr.2. **(B)** The gene *TMS5* mutates at cds.71 in T98S with a transversion from cytosine (C) to adenine (A).

Following the BSA strategy, two F_2_ bulked DNA samples from T98S/R996 were used to rough map the *tms(t)* responsible for T98S on chr.2 with the help of the SSR markers RM11267 and RM12992 (**Figure 1A**). We sampled 1,334 F_2_ TGMS individuals to fine-map *tms(t)* within 44 Kb between sequence tag site (STS) markers s6-6 and s6-17 corresponding to positions 6,387,223–6,429,700 in the referenced Nipponbare (NPB) genome (**Table S1**). This region carried 11 genes, and incidentally, the gene *TMS5* (LOC_Os02g12290.1, labeled as Gene5) controlling the TGMS trait was located on 6,397,342–6,399,236 (**Figure 1A**). The *TMS5* in NPB genome has six exons and five introns and a 909 bp CDS encoding a product of nuclear ribonuclease Z. Through sequencing *TMS5*, we found that only cds.71 at 6,397,412 mutated with a transversion from cytosine (C) to adenine (A) in T98S (**Figure 1B**). Such an alteration may enable the production of a stop codon (TAG) at cds.70–72, terminating transcription. To our surprise, the point of cds.71 was where the original *tms5* mutants AnS and ZhS had mutated (Zhou et al., 2014), suggesting that *TMS5* is the candidate gene responsible for T98S.

### *tms5* Is Responsible for T98S in Fertility

**Figure 2:**
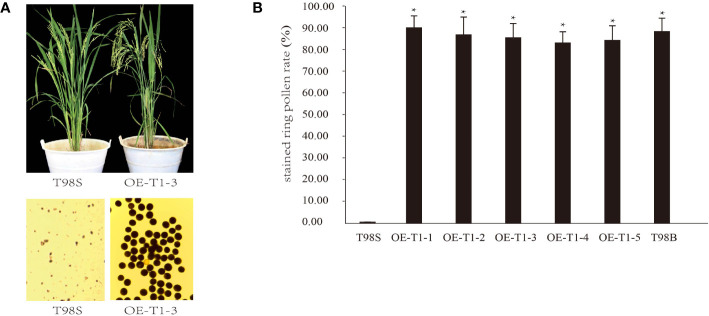
Genetic complementary analysis of *TMS 5* for T98S. **(A)** Phenotype of pollen fertility by over-expressing the coding sequence (CDS) of *TMS5* in T98S. **(B)** Difference in stained ring pollen rate between T98S (as a control) and transgenic rice. The five T1 transgenic lines (OE-T1-1~ OE-T1-5) grown under high temperature were all recovered in pollen fertility. * indicates a significant difference at *P* < 0.05.

A complementary experiment was performed to validate whether *tms5* is responsible for T98S. By *A. tumefaciens*-mediated transformation, a pTMS5 construct overexpressing full-length CDS of *TMS5* (cloned from T98B) was introduced into T98S. The 5 T_1_ lines including OE-T1-1~ OE-T1-5 were detected positive by PCR (**Figure S1**). They were grown in the paddy field and exposed to a high temperature of ~ 29°C during a sensitive fertility period. During this period, T98S at heading remained completely sterile with no stained ring pollen grains. However, all transgenic lines that recovered their pollen fertility had an average stained ring pollen rate of 86.53%± 6.32%, similar to the ones of T98B (89.37% ± 5.27%) (**Figures 2A, B**). These results support that T98S was actually a *tms5* line.

### T98S Originated Directly From T98B

**Figure 3:**
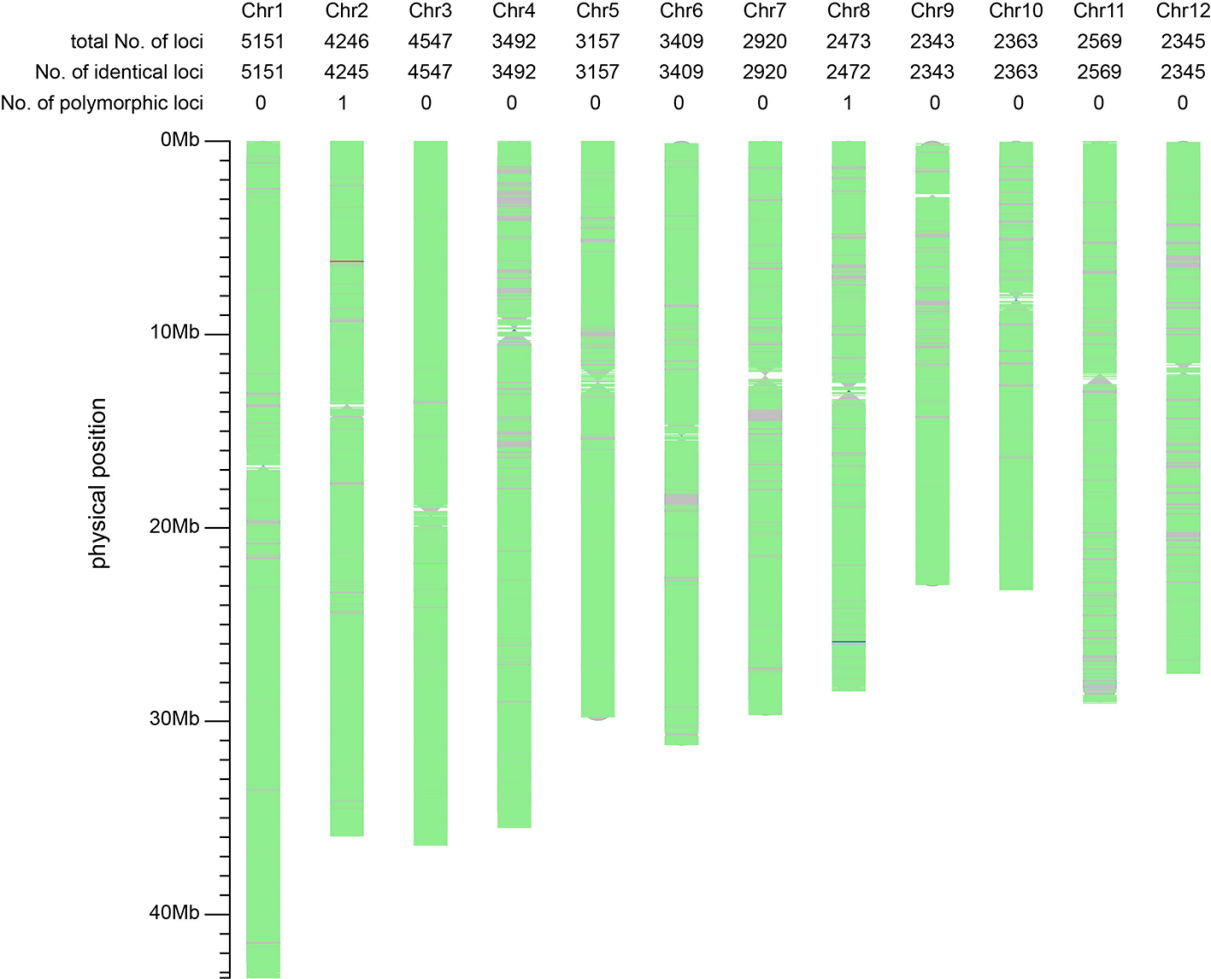
Comparative analysis of the background between T98S and T98B using 39,015 SNPs on Rice 56K SNP Array. The regions with no marker are represented using grey color. The green areas indicate the non-polymorphic SNPs (identical loci) located on. The red line and the blue line represent a homozygous-type and a heterozygous-type polymorphic locus on 6,211,844 chr.2, and 25,891,109 chr.8. The genetic similarity between T98S and T98B amounts to 99.99% (39,013/39,015), suggesting T98S directly mutates from T98B.

We were interested in whether the mutation of T98S at cds.71 on *tms5* originated directly from T98B or was transferred by another *tms5* line. To explore this, the genetic difference between T98S and T98B was assessed on Rice 56K SNP Array using Nipponbare as a reference. The two samples were tested with three replicates using 39,193 SNPs, achieving a repetition rate of 99.93% for T98S and 99.60% for T98B. The homozygosity rate was found to be 99.15% (38,832/39,165) in T98S and 99.14% (38,701/39,036) in T98B (**Table 1**). Later, a total of 39,015 qualified SNPs covering the whole genome (with a density of ~105 per 1 Mb) were selected to evaluate their similarity. It was estimated that 39,013 SNPs (accounting for 99.99% of total SNPs) showed no polymorphism between T98S and T98B. The remaining two SNPs differed in being homozygous on 6,211,844 chr.2 and heterozygous on 25,891,109 chr.8 (**Figure 3**). This suggested that T98S was a true sibling of T98B.

**Table 1:** Analysis of repetition rate and homozygous degree for T98B and T98S.

Line	TotalSNP	Repeatable SNPs	UnrepeatableSNPs	Repetition rate	Homozygous SNPs	HeterozygousSNPs	Homozygousdegree
T98B	39,163	39,036	157	99.60%	38,701	335	99.14%
T98S	39,193	39,165	28	99.93%	38,832	333	99.15%

### T98S Can Be Distinguished From AnS and ZhS

**Figure 4:**
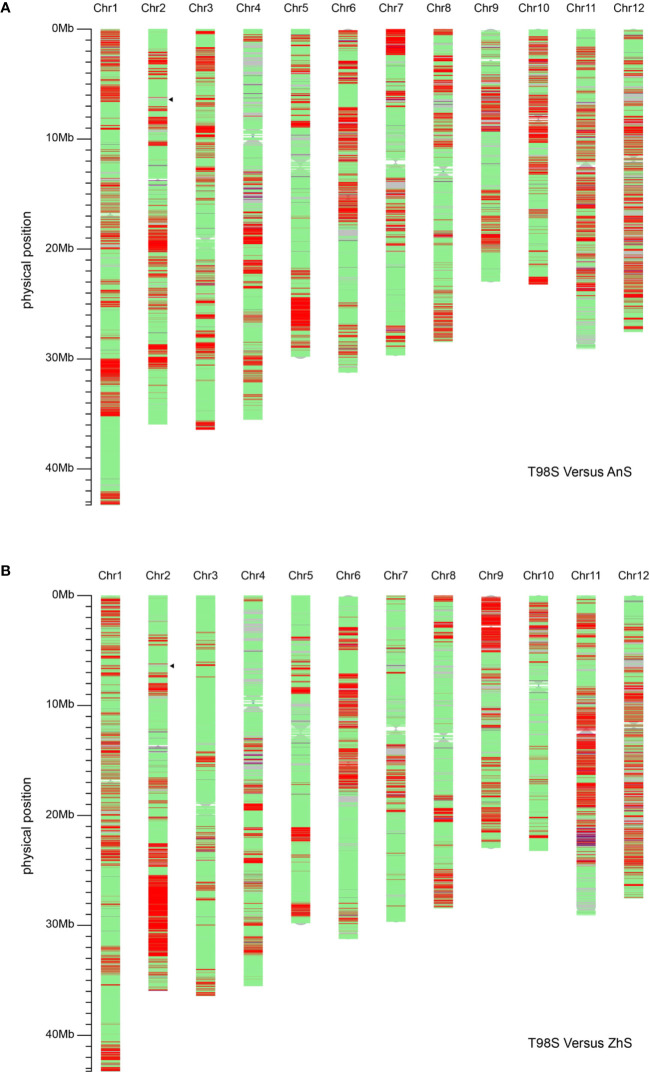
Analysis of the background of T98S compared with AnS **(A)** and ZhuS **(B)** using 39,165 single nucleotide polymorphisms (SNPs) on Rice 56K SNP Array. The green areas indicate the non-polymorphic SNPs located on. The red areas cover homozygous-type polymorphous SNPs, and the blue areas cover heterozygous-type polymorphous SNPs. The position that the black triangle points toward locates *TMS5*.

In addition, we compared T98S with two original *tms5* lines, namely AnS and ZhuS, using 39,165 SNPs. The difference rate was 27.16% (10,638/39,165) between T98S and AnS, and 22.53% (8,825/39,165) between T98S and ZhS (**Figure 4**, **Table 2**). The polymorphous SNPs covered 12 chromosomes. T98S differed greatly from AnS on chr.12 (36.45%), chr.11 (32.27%), and chr.6 (30.33%), and on chr.11 (39.25%), chr.9 (37.10%), and chr.12 (34.97%) for ZhS. On chr.2, where *TMS5* is located, T98S could distinguish AnS and ZhS at a difference rate of 25.80% and 34.44%, respectively (**Figure 4**, **Table 2**). These data indicate that T98S has a different genetic background from AnS and ZhuS.

**Table 2:** Background analysis of T98S compared with AnS and ZhuS.

Chromosome	Total number	T98S versus ANS			T98S versus ZhS		
		Non-polymorphism SNP number	Polymorphous SNP number	Difference rate	Non-polymorphism SNP number	Polymorphous SNP number	Difference rate
1	5,167	3,775	1,392	26.94%	3,888	1,279	24.75%
2	4,260	3,161	1,099	25.80%	2,793	1,467	34.44%
3	4,563	3,386	1,177	25.79%	4,143	420	9.20%
4	3,505	2,680	825	23.54%	2,925	580	16.55%
5	3,170	2,404	766	24.16%	2,714	456	14.38%
6	3,419	2,382	1,037	30.33%	2,609	810	23.69%
7	2,930	2,225	705	24.06%	2,619	311	10.61%
8	2,481	1,857	624	25.15%	2,000	481	19.39%
9	2,353	1,711	642	27.28%	1,480	873	37.10%
10	2,371	1,695	676	28.51%	2,063	308	12.99%
11	2,581	1,748	833	32.27%	1,568	1,013	39.25%
12	2,365	1,503	862	36.45%	1,538	827	34.97%
Sum	39,165	28,527	10,638		30,340	8,825	
Average				27.16%			22.53%

We focused on the area surrounding *tms5* to identify the specific SNPs for T98S. The number of SNPs differing between T98S and AnS (or ZhS) was one in the upstream area (5.39–6.39 M) and 15 in the downstream area (6.39–7.39 M), and they were concentrically distributed on the region from 7.03 M to 7.38 M (**Figure 4**, **Table 3**). Although AnS and ZhS could be characterized by 13 SNPs located from 5.79 Mb to 6.84 Mb through genome resequencing (Yan et al., 2019), there were no polymorphous SNPs to distinguish them through microarray analysis.

**Table 3:** The single nucleotide polymorphisms (SNPs) within 1.0 Mbp up- and down-stream around TMS5 among three thermo-sensitive genic male sterility (TGMS) lines referenced as Nipponbare.

SNP ID	Position*	T98S	AnS	ZhS
AX-155464611	6,211,844	C/C	T/T	T/T
AX-165095954	7,032,362	T/T	C/C	C/C
AX-165089120	7,044,610	C/C	T/T	T/T
AX-115808897	7,072,374	A/A	G/G	G/G
AX-154242679	7,119,202	G/G	A/A	A/A
AX-154944191	7,132,042	A/A	C/C	C/C
AX-165092511	7,238,480	A/A	G/G	G/G
AX-115797125	7,248,666	T/T	C/C	C/C
AX-165103200	7,282,914	C/C	T/T	T/T
AX-154410183	7,289,955	C/C	T/T	T/T
AX-155524733	7,299,368	A/A	G/G	G/G
AX-165103287	7,324,667	T/T	C/C	C/C
AX-115755253	7,357,661	T/T	C/C	C/C
AX-165095546	7,359,341	C/C	T/T	T/T
AX-165094219	7,366,395	G/G	A/A	A/A
AX-154929815	7,376,951	G/G	A/A	A/A

*The position is referenced as the Nipponbare genome.

### An Indica-Preferred TGMS Trait May Have Resulted From an Unbalanced Allele Frequency of Cds.70

**Figure 5:**
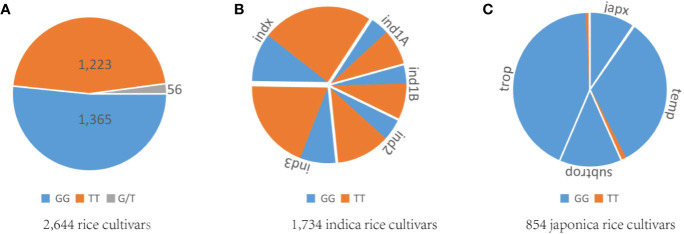
Analysis of allele frequency at cds.70 on *TMS5*. **(A)** Genotype distribution of GG, TT, and G/T at cds.70 for 2,644 rice cultivars. **(B)** Allele frequency of GG and TT at cds.70 for 1,734 indica rice cultivars. **(C)** Allele frequency of GG and TT at cds.70 for 854 japonica rice cultivars. These samples are indica or japonica and were collected from the Rice SNP-Seek Database. The two genotypes at cds.70, GG and TT, showed unbalanced frequency between subspecies. The TT is overwhelming in indica group, while GG is preferred by the japonica group.

**Figure 6:**
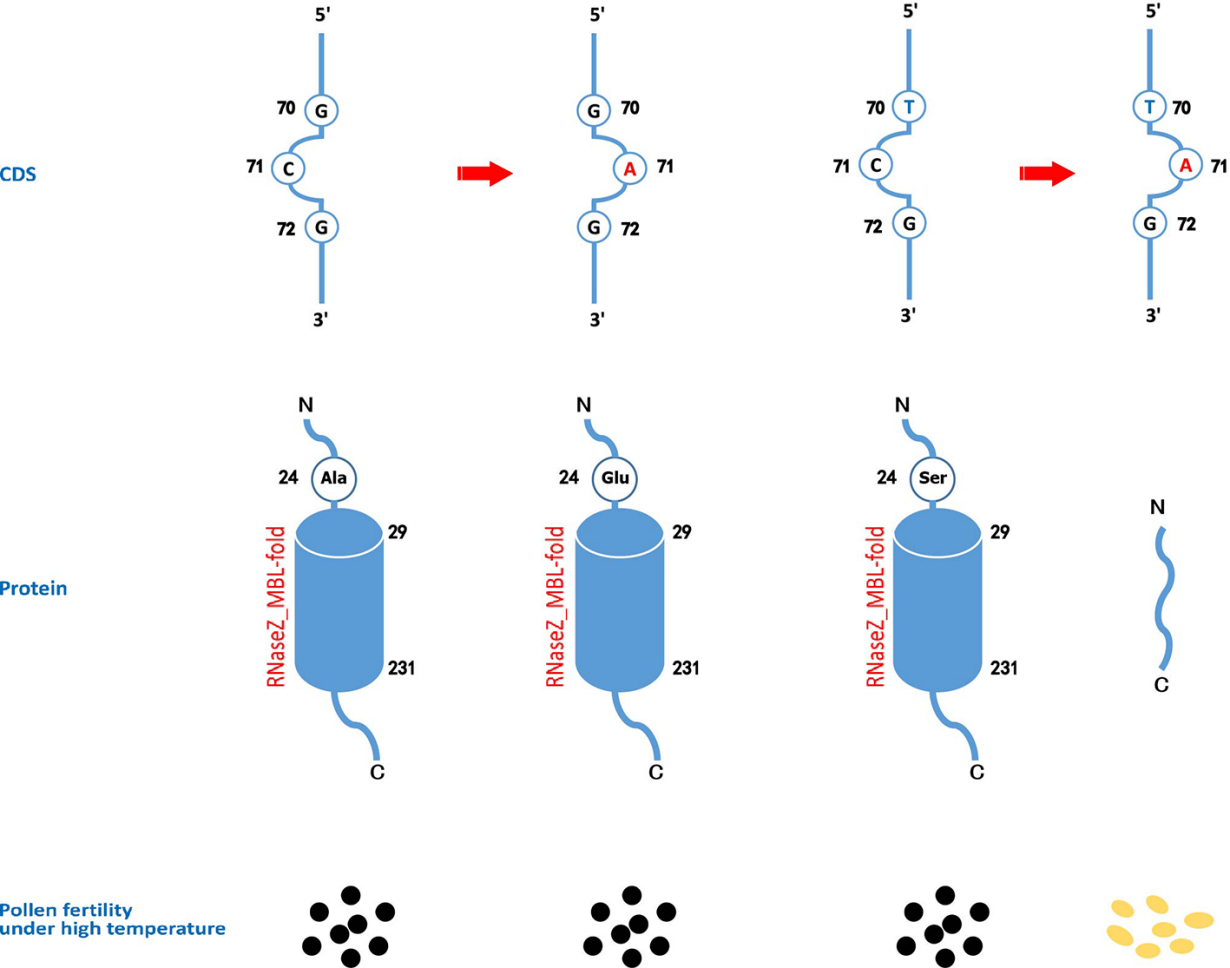
The effects on the protein domains and pollen fertility of different genotypes of cds.70 combined with cds.71 on *TMS5*. A functional TMS5 provides the necessity of pollen development. Though CD-SEARCH analysis, TMS5 has a conserved domain of RNase Z_MBL-fold regarding 29-231 amino acid residues. When cds.71 mutates from C to A, the cds.70(G) leads to a replacement of alanine (Ala) by glutamate (Glu) on the 24^th^ amino acid residue that has no impact on the conserved domain. However, the cds.70(T), if combined with cds.70(A), will induce a stop codon at the 24^th^ site, thus resulting in a function-loss RNase Z so as to gain a thermo-sensitive genic male sterility (TGMS) trait.

Rice cultivars in China and Asia are divided into two major *O. sativa* subspecies: indica and japonica. Less is known about why all original *tms5* mutants were found in the indica background. Hence, we focused on comparing the difference in the genotype at cds.70–72 between the two subspecies in Rice SNP-Seek Database. In this database, 2,644 rice cultivars were defined as indica or japonica. Of these, 1,789 were indica (consisting subpopulations indx, ind1A, ind1B, ind2, and ind3) and 855 were japonica (consisting subpopulations jap, temp, subtrop, and trop) (**Table S2**). We found that cds.70 existed as a G/T haplotype, while no variation was detected on cds.71 and cds.72 (**Figure 5**; **Table S2**). Conserved domain analysis by CD-search showed that the effects on RNase Z differed between the two alleles of cds.70 combined with cds.71 when it mutates from C to A. It revealed that cds.70 (T) along with cds.71 (A) generated a stop codon (TAG) at cds.70–72 to lose a conserved domain inducing a TGMS trait (**Figure 6**). In contrast, cds.70 (G) only led to a replacement of alanine (Ala) with glutamate (Glu) on the 24^th^ amino acid residue, and therefore conserved domain Z_MBL-fold (amino acid residues 29–231) of RNase remained unaffected and so did the normal pollen development (**Figure 6**). Interestingly, in the 2,588 cultivars with a homozygous state at cds.71 (1,365 for GG and 1,223 for TT), we noticed an unbalanced allele frequency between the two subspecies. In indica rice, cds.70 (T) reached a higher mean frequency of 67.52%, with 66.67% for indx, 62.68% for ind1A, 63.90% for ind1B, 70.53% for ind2, and 70.53% for ind3 (**Table 4**, **Figure 5B**). On the contrary, the allele frequency decreased dramatically in the japonica cultivars, with an average level of just 1.75%, with 2.41% for japx, 2.78% for temp, 0.00% for subtrop, and 1.34% for trop (**Table 4**, **Figure 5C**). Therefore, the biased allele frequency of cds.70 would have led to an indica-preferred TGMS trait.

**Table 4:** Differences in allele frequency of cds.70 on *TMS5* between indica rice and japonica rice.

Subspecies	Subpopulation	Total number	GG		TT		G/T	
			Number	Frequency	Number	Frequency	Number	Frequency
indica		1,789	526	29.40%	1208	67.52%	55	3.07%
	indx	594	159	26.77%	410	69.02%	25	4.21%
	ind1A	204	68	33.33%	126	61.76%	10	4.90%
	ind1B	196	68	34.69%	122	62.24%	6	3.06%
	ind2	280	74	26.43%	201	71.79%	5	1.79%
	ind3	469	125	26.65%	335	71.43%	9	1.92%
japonica		855	839	98.13%	15	1.75%	1	0.12%
	japx	81	79	97.53%	2	2.47%	0	0.00%
	subtrop	112	112	100.00%	0	0.00%	0	0.00%
	trop	368	363	98.64%	5	1.36%	0	0.00%
	temp	288	279	96.88%	8	2.78%	1	0.35%

## Discussion

In the last three decades, nearly 20 original P/TGMS mutants have been identified (Fan and Zhang, 2018). They display diverse sensitivity against day-length/temperature. The japonica PGMS mutant Nongken58S can be converted from fertility to sterility when the day-length is longer than 14 h (Zhang et al., 1987), while another japonica mutant *csa* (for carbon starved anther) showed male sterile under short-day conditions with a critical photoperiod of 12.5–13.0 h (Zhang et al., 2012). Furthermore, the Nongken58S derivatives were also noticed to be influenced by temperature in fertility alteration (Zhang et al., 1992), indicating the existence of a compensating mechanism between critical day-length and temperature. The original TGMS mutants were almost discovered in indica rice. The ones including 5460S (Wang et al., 1995), Hengnong S-1 (Qi et al., 2014), AnS-1 (Chen et al., 1994), ZhS (Sheng et al., 2013), XianS (Peng et al., 2010), and Mian 9S (Huang et al., 2008) became sterile when the temperature was beyond 24°C, while J207S and go543S had completely abortive pollen grains below 31°C (Jia et al., 2001; Liu et al., 2010).

The diverse phenotypes were regulated *via* different genetic mechanisms. The genes *PMS3* and *PMS1T*, two major loci responsible for Nongken58S, both encode long noncoding RNA (lncRNA). The decreased level of *PMS3* together with the increased level of *PMS1* in transcription leads to male sterility under long-day conditions in Nongken58S (Ding J. H. et al., 2012; Fan et al., 2016). A single-locus *TMS5* encoding RNase Z controls the fertility of AnS. In a normally fertile line, *TMS5* itself is insensitive to temperature, but several targets of RNase Z such as ubiquitin-60S ribosomal protein L40 family (Ub_L40_) members Ub_L40_1, Ub_L40_2, and Ub_L40_4 dramatically accumulated their mRNA levels in AnS under high temperature.

It is generally accepted that AnS and ZhS are the two original donors for the TGMS lines in practice in China. Through map-based cloning, Zhou et al. (2014) and Sheng et al. (2013) demonstrated that ZhS is another *tms5* mutant allele to AnS, but surprisingly, they are commonly subjected to the critical point of cds.71 altered from C to A. Because *tms5* gene is very easy for breeders to transfer from one rice cultivar to another, we speculated if AnS and ZhS were derived from a common donor or differed in sources. To explore this, Yan et al. (2019) investigated their genetic differences by genome resequencing and sought 13 polymorphous SNPs around *tms5* to distinguish the AnS-derivatives from the ZhS-derivatives.

From such observations we hypothesized the cds.71 may act as a mutation hotspot to induce a TGMS trait. The most convincing evidence was that many original *tms5* mutants were found. In fact, several mutants like XianS, Q523S, Q524S, N28S, G421S, and Q527S were also revealed to be controlled by *tms5* (Peng et al., 2006; Peng et al., 2010; Zhou et al., 2014). However, there were no details to verify their true identity, as it is possible for a fake mutant to be unknowingly selected in the paddy due to biological confounding. One example is a well-known TGMS line named “Guangzhan63S”. According to Yang’s description, Guangzhan63S was selected by a crossing between N422S (a Nongken58S derivative) and Guangzhan63 (Yang et al., 2002). However, it was finally proven to be a *tms5* line by molecular analysis (Xu et al., 2011; Zhou et al., 2014). So, in this sense, it was important for us to verify the true identity of the TGMS mutant T98S. Previous works found T98S featured a rapid stability and showed quite similar morphological traits as T98B (Tan et al., 2018). Our work herein demonstrated that T98S is genetically a real sibling of T98B (**Figures 3** and **4**, **Table 2**) and another *tms5* mutant with a C-to-A transvertion at cds.71 (**Figures 1** and **2**). Coincidentally, we also reported another TGMS mutant “BM2-23” (also irradiation-induced from T98B) that was found to be allele to T98S (Tan et al., 2018).

The cds.71 may tend to mutate and that led us to question why the original *tms5* mutants cannot be found in japonica rice. The TGMS trait is noticeable and the period for selection lasts for approximately three months, and so it is impossible to go unobserved. This biased disappearance may be related to the effects of genotype of cds.70-72. We noticed a T/G haplotype existed at cds.70 and the allele frequency was opposite between indica rice and japonica rice (**Figure 5**). Therefore, the T rather than G at cds.70 resulted in that TGMS trait (**Figure 6**). The typical Chinese early-season indica cultivars (e.g., XIANGZAOXIAN7, ZAOXIAN14, and ZAOXIAN240) in the “ind1A” subpopulation always have T at cds.70 in Rice SNP-Seek Database, and it may partly the reason why AnS, ZhS, and T98S were found in early-season indica rice; whereas in japonica rice, only 1.75% cultivars carry a T at cds.70 (**Figure 5**), so it would decrease the probability to originate a *tms5* mutant. Particularly, the subspecies-based preference provides an alternative for developing TGMS lines in the breeding. For example, mutagenesis with chemical or physical treatment would be an effective way to obtain an indica *tms5* line (Tan et al., 2018). For japonica rice, the *tms5* lines are unlikely to be induced *via* mutagenesis, and the possible way will be transferring *via* an indica *tms5* line (Deng, 2005) or gene editing on *TMS5* (Zhou et al., 2016). We also did not find a C-to-A transvertion at cds.71 in fertile rice, and we believe the likely cause is a certain specific locus (like cds.70.(G)) that inhibits its ability to mutate.

The mechanisms underlying gene mutation are extremely complex. A locus in a gene may be conserved or vary in different ways and at different frequencies. For example, the loci at an intron vary more frequently than those at splicing sites in *Arabidopsis thaliana* (Brown, 1996); G/C bases are commonly found to be mutated in RGYW/WRCY on AID (encoding activation-induced cytosine deaminase) (Muramatsu et al., 1999). In patients with breast-ovarian cancer, the gene *BRCA2* has a high frequency of the “AAGA” deletion at cds.1310-1313 (Laarabi et al., 2017). To date, few mutation hotspots have been reported in rice. Interestingly, regardless of being spontaneous (like AnS), induced by gamma irradiation (like T98S) or genetically recombined by fertile line crossing (like ZhS), the *TMS5* tended to mutate at cds.71 with a C-to-A transvertion. However, we are not sure whether TCG is more easily mutatable in other position. Any gene can be known to potentially have hotspots if it induces a noticeable trait such as the one found here in the paddy. Additionally, local DNA sequences were found to strongly influence mutation frequency. In the *lacI* gene of *Escherichia coli*, several spontaneous deletions were caused by DNA repeats with specific termini of palindromic sequences (Farabaugh et al., 1978), and the hotspots with a C-to-T substitution may result from the spontaneous deamination of 5-methylcytosine (Coulondre et al., 1978). Sometimes, the mutation frequency was found to be closely correlated with the level of transcription mediated by regulatory elements (Yoshikawa et al., 2002). For exploring the mechanisms underlying hotspots in higher plants, the cds.71 on *TMS5* would be an ideal locus to focus on for future fundamental studies.

## Conclusion

The *tms5*-based two-line hybrid rice has greatly contributed to increasing the cereal supply and is going to be expanded to a larger scale in China and abroad. However, our understanding of *TMS5* is still limited. In this study, we identified another *tms5* mutant T98S and investigated the behavior of the cds.70–72 on *TMS5*. By detecting the genetic background and using map-based cloning, we demonstrated that T98S originated independently from T98B, and cds.71 was found to be the mutation point responsible for T98S. Furthermore, the discovery of an unbalanced allele frequency of cds.70 partly offers an explanation for an indica-preferred TGMS trait. These findings improve our understanding of *tms5* and provide new approaches for developing *tms5* indica lines.

## Data Availability Statement

The raw data supporting the conclusions of this article will be made available by the authors, without undue reservation, to any qualified researcher.

## Author Contributions

YT designed the study, performed all experiments, summarized the results, and wrote the manuscript. DY and MD directed and supervised all experiments. XS, BF, XS, and LL participated in the work of map-based cloning. ZS, DY, and HL detected the genetic background. ZL analyzed the allele frequency. All authors contributed to the article and approved the submitted version.

## Funding

This study was supported by the Agriculture Science and Technology Innovation Program of HHRRC and HAAS, China (Grant No. YB201907 and 2017XC09), the Natural Science Foundation of Hunan, China (Grant No. 2019JJ40206), the Hunan Science and Technology Talents Support Project (Grant No. 2019TJ-Q08) and the National Key Research and Development Program of China (Grant No. 2016YFD0101101).

## Conflict of Interest

The authors declare that the research was conducted in the absence of any commercial or financial relationships that could be construed as a potential conflict of interest.

## Supplementary Material

Figure S1 The PCR amplification of T1 transgenic lines over-expressing the cds of *TMS5*. M, DNA marker; 1, T98S(negative control); 2. pTMS5 (positive control); 3-7, transgenic lines referring to OE-T1-1, OE-T1-2, OE-T1-3, OE-T1-4 and OE-T1-5. The samples were detected by PCR using the specific primer ubtsm-F/R.

Table S1 The primers used in this study. *The position is referenced as the Nipponbare genome.
