# Supplementary figures and images for: The Cds.71 on TMS5 May Act as a Mutation Hotspot to Originate a TGMS Trait in Indica Rice Cultivars
Source: Front Plant Sci. 2020 Aug 7;11:1189. doi: 10.3389/fpls.2020.01189 (PMC7427412; doi:10.3389/fpls.2020.01189)

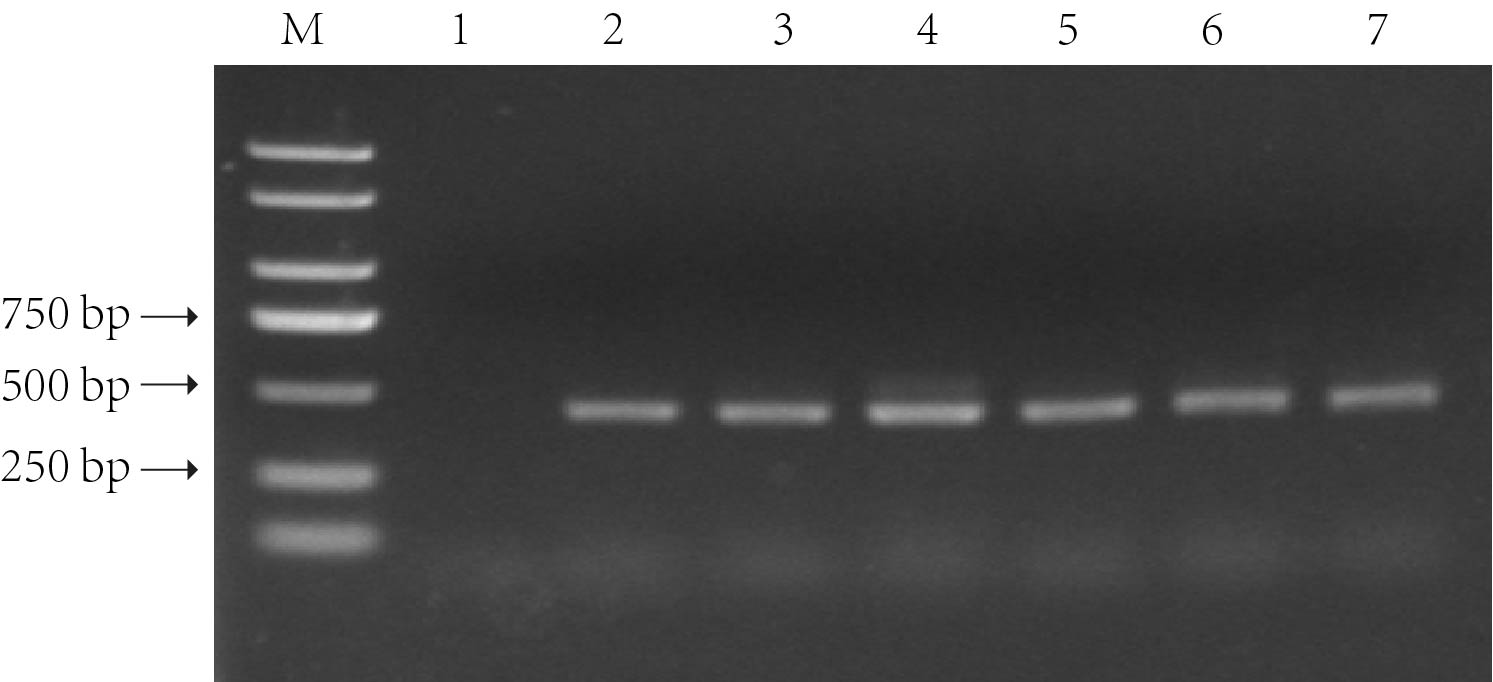

Supplement: Figure S1 — The PCR amplification of T1 transgenic lines over-expressing the cds of TMS5. M, DNA marker; 1, T98S(negative control); 2. pTMS5 (positive control); 3-7, transgenic lines referring to OE-T1-1, OE-T1-2, OE-T1-3, OE-T1-4 and OE-T1-5. The samples were detected by PCR using the specific primer ubtsm-F/R. [file Image_1.jpeg]
