# Supplementary material for: The Cds.71 on TMS5 May Act as a Mutation Hotspot to Originate a TGMS Trait in Indica Rice Cultivars
Source: Front Plant Sci. 2020 Aug 7;11:1189. doi: 10.3389/fpls.2020.01189 (PMC7427412; doi:10.3389/fpls.2020.01189)
Supplement: Table S1 — The primers used in this study. *The position is referenced as the Nipponbare genome. [file Table_1.doc]

**Supplemental Table S1 |** The primers used in this study

| Primer | Sequence (5’-3’) | Length of PCR product  (bp) | Location on chr.2* |
| --- | --- | --- | --- |
| RM12667 | F: GGCGAGACGACTGCTCTACTGC | 153 | 5555391..5555543 |
|  | R:GACGTTGTCGAACACGATGAGC |  |  |
| RM12992 | F: CGGCTTCAGGAACTTCACCATCG | 146 | 10701387..10701532 |
|  | R:CTCCGCGAACCCTAACACCATCC |  |  |
| Ind6.03 | F:CGAACTTGACTGTGTATAGT | 254 | 6030987..6031240 |
|  | R:AGTTCGGTTCGAGCTTGTTC |  |  |
| Ind6.62 | F:CTTGAGCCAAACTTTCTC | 139 | 6623950..6624088 |
|  | R:CAATTTCCTGGCGATGGA |  |  |
| s6-6 | F:ATCTGACAACAATCTGCATG | 310 | 6387223..6387532 |
|  | R:GACAGCCTTAGTTAGTGAGT |  |  |
| s6-11 | F:CTTGAGCCTCCAGCAATG | 442 | 6405442..6405883 |
|  | R:ATATGCATGCCAATAGAAC |  |  |
| s6-17 | F:GCTTTAACAGTATACATCAT | 384 | 6429317..6429700 |
|  | R:TTGTTGTGTACAGTTCGATC |  |  |
| ubi/tms | ubi-F:CTTTTGTCGATGCTCACCCTG | 463 |  |
|  | tms-R:TTGTGGCTGAGCTCGGACT |  |  |

*The position is referenced as the Nipponbare genome.
